# Nutritional adequacy of a cows’ milk exclusion diet in infancy

**DOI:** 10.1186/s13601-016-0109-8

**Published:** 2016-06-02

**Authors:** Kate Maslin, Erin M. Oliver, Karen S. Scally, Josh Atkinson, Keith Foote, Carina Venter, Graham Roberts, Kate E. C. Grimshaw

**Affiliations:** School of Health Science and Social Work, University of Portsmouth, Portsmouth, UK; Clinical and Experimental Sciences and Human Development in Health Academic Unit, Faculty of Medicine, University of Southampton, Southampton, SO16 6YD UK; Faculty of Social and Human Sciences, 58/2113, University of Southampton, Highfield, Southampton, SO17 1BJ UK; Food Standards Agency, London, UK; Hampshire Hospitals Foundation Trust, Winchester, UK; Cincinnati Children’s Hospital Medical Center, 3333 Burnet Avenue, MLC7028, Cincinnati, OH 45229 USA; NIHR Respiratory Biomedical Research Unit, University Hospital Southampton NHS Foundation Trust, Southampton, SO16 6YD UK; Department of Nutrition and Dietetics, Southampton Children’s Hospital, Southampton, SO16 6YD UK

**Keywords:** Cows’ milk allergy, Dietary exclusion, Nutritional intake, Infant

## Abstract

**Background:**

Infants with suspected cows’ milk allergy are required to follow a strict milk exclusion diet which may lead to nutritional deficiencies, especially if not supervised by a healthcare professional. The aim of this study was to assess the nutritional adequacy of a cows’ milk exclusion diet in a group of UK infants over a period of 6 months.

**Methods:**

Participants in this study are a subgroup of the Prevalence of Infant Food Allergy study, a prospective food allergy birth cohort study from the South of England. Each infant consuming a milk free diet, following advice from a specialist allergy dietitian, was matched to two control infants who were consuming an unrestricted diet, forming a nested matched case–control study. Detailed food diaries completed prospectively for 1 week per month over a 5 month period, were coded and analysed according to a standard protocol.

**Results:**

The diets of 39 infants (13 milk-free and 26 controls) were assessed. Mean age at diet commencement was 14 weeks. Two of the eleven infants started on an extensively hydrolysed formula did not tolerate it and required an amino acid formula for symptom resolution. All infants had mean intakes in excess of the estimated average requirement for energy and the recommended nutrient intake (RNI) for protein, calcium, iron, selenium, zinc, vitamins A, C and E. Vitamin D intake was in excess of the RNI at all time-points, except at 44 weeks of age. Across the study period, selenium intake was higher for infants consuming a milk free diet whilst vitamin C intake was higher for infants consuming an unrestricted diet. Differences were found between the two groups for protein, calcium, iron and vitamin E intakes at differing time points.

**Conclusion:**

This study demonstrated that although infants consuming a milk-free diet have a nutritional intake that is significantly different to matched controls who are eating an unrestricted diet, this difference is not constant and it is not seen for all nutrients. Further research in infants without dietetic input is needed to explore the nutritional implications of unsupervised cows’ milk exclusion diets.

## Background

Cows’ milk allergy (CMA) is the most common infant food allergy with an estimated prevalence of 1.26–2.9 % in the UK [1, 2], the majority of which is non-IgE mediated [3]. Parents of reactive children are advised that their child should follow a special weaning diet avoiding all forms of cows’ milk until the allergy is outgrown. This avoidance should ideally be supported by input from an allergy dietitian to monitor and optimise the nutritional content of the diet and to maintain potential growth [4, 5].

It is thought that perceived food allergy could be ten times higher than that confirmed by appropriate tests [6]. This is particularly the case in paediatric food allergy, where parents may incorrectly perceive their child to have experienced an adverse reaction to a food [7]. With allergy services considered inadequate to meet demand in many countries [8]^,^ unwarranted exclusion diets are often initiated by parents [9–12]. This heightens the likelihood of unsupervised exclusion diets at a time in life that is critical for growth, development and establishment of eating habits.

Adequate nutritional intake in infancy is essential to ensure appropriate physiological and mental development [13]. Exclusion of any food group can result in a nutritionally deficient diet, but the elimination of dairy in infancy is particularly likely to cause nutritional deficiencies [14]. This is highly significant as both reduced dietary variety [15, 16] and deficiencies of specific micronutrients [17] are postulated to be implicated in food allergy development. Exclusion diets, in particular cows’ milk exclusion diets, have been associated with poor growth in childhood [18, 19].

Studies from various countries have investigated the nutritional intake of children consuming an exclusion diet secondary to cows’ milk and other food allergies, demonstrating differences in both macro and micro nutrient intakes [20–30]. However, most of the previous literature in this area is cross sectional. Since the assessment of dietary intake during infancy is complicated by changing development and food refusal [31], a snapshot of dietary intake is unable to accurately represent the changing infant diet. This study will compare the dietary intake of infants consuming a cows’ milk exclusion diet for CMA to those consuming an unrestricted diet, with the aim of assessing adequacy of micro and macronutrient intake over a period of 20 weeks.

## Methods

### Overview of birth cohort study

The data reported in this paper consists of a sub group of infants who were recruited as part of a prospective birth cohort study. The Prevalence of Infant Food Allergy (PIFA) study, the UK arm of the EuroPrevall project [32], recruited 1140 infants between 2006 and 2008 in the Southampton/Winchester area in the South of England. Infants were followed up to 2 years of age in order to assess the prevalence and natural history of food allergies.

### Data collection

As part of the study, parents kept prospective food diary data. Food diaries were completed until the age of one and returned every 4 weeks [33, 34]. Every fourth week the diaries were more detailed which allowed the infants macro and micronutrient intake to be calculated.

### Dietetic support

Infants suspected of having an adverse reaction to cows’ milk were given advice to follow a cows’ milk exclusion diet to determine if their symptoms resolved. The advice, given by a specialist allergy dietitian, detailed strict and complete cows’ milk avoidance, with accompanying written information and details of milk-free products and recipes provided. Advice was provided to avoid other mammalian milk and milk products (e.g. sheep, goat) as there is known cross reactivity with cows’ milk [35]. These infants were not excluding any other foods from their diet (e.g. soya). If symptoms improved on the exclusion diet, the infant continued with the diet and were termed “milk-free”. Children who did not report an adverse reaction to cows’ milk did not receive any dietetic input.

### Selection of participants

Each infant following a milk exclusion diet who had returned at least 3 weeks of quantitative diet data covering a period of 12 weeks had their dietary intake data analysed. Each reactive infant was matched to two control infants (who were consuming an unrestricted diet for their age), according to age, number of food diaries available and breastfeeding status, thus forming a nested matched case–control study.

### Dietary analysis

Dietary analysis was performed with the dietary analysis package ‘CompEatPro’ (Nutrition Systems, 2008). Breast milk intake was estimated by age using average values obtained from previous published literature [36, 37]. Portion sizes were recorded in household measures and converted into weights using published data or by weighing the stated portion-size for that food. Food diaries were coded according to a standard protocol by two nutritionists and a dietitian. To ensure the most data was available for the RM-ANOVA, diaries 6–11 (24–44 weeks of age) were analysed.

### Statistical analysis

Mean daily values for nutrient intake were calculated by the dietary analysis package, imported into Statistical Package for the Social Sciences version 18 (SPSS Inc) and compared to UK Recommended Nutrient Intakes (RNI) [38]. A General Linear Model Repeated Measures analysis of variance with between subject factors (RM-ANOVA) was carried out to determine whether there was a difference in dietary intake between the groups for macronutrients and selected micronutrients. Specific time point analyses were carried out post hoc.

### Ethical, consent and permissions

The North and Mid Hampshire Local Research Ethics Committee approved the study protocol (reference O5/Q1703/34). Written consent was provided for each participant by their parent/guardian.

## Results

### Participant characteristics

In total 74 infants were required to follow a milk free diet as part of the birth cohort study. Of the 74 infants, 13 infants met the inclusion criteria to have at least 3 quantitative diaries collected over 12 weeks available for analysis.

Mean age of infants at diet commencement was 14 weeks (range 5–36 weeks). Each milk-free infant was matched to 2 control infants, resulting in dietary analysis of 13 milk-free and 26 control infants. Baseline characteristics are detailed in Table 1.

**Table 1:** Baseline characteristics of participants

	Milk free group (n = 13)	Control group (n = 26)	p
Caucasian ethnicity	12 (92.3)	26 (100)	0.333^†^
Female sex	4 (30.7)	11 (42.3)	0.728^†^
Mothers’ mean age, years	32.0	32.4	0.872^^^
Fathers’ mean age, years	34.2	34.9	0.988^^^
Highest education of parents			0.598^§^
Low (up to 12 years)	3 (23)	8 (30.7)	
Intermediate (>12 years, e.g. college)	5 (38.5)	6 (23)	
High (e.g. university)	5 (38.5)	12 (46.1)	
Allergies in family			
Maternal atopy (A, AR or E)*	11 (84.6)	16 (61.5)	0.269^^^
Paternal atopy (A, AR or E)*	7 (53.8)	16 (61.5)	0.736^^^
Maternal food hypersensitivity	2 (15.4)	3 (11.5)	1.000^^^
Paternal food hypersensitivity	2 (15.4)	7 (26.9)	0.689^^^
Urban living environment	2 (15.4)	2 (7.7)	0.589^†^
Mean number of siblings	0.6	0.3	1.000^^^
Mean birth weight (g)	3538	3476	0.738^^^
Mean duration breastfeeding (months)	1.75	2.68	0.189^^^
Ever breastfed	7 (53.8)	22 (57.8)	0.742^†^

Data are expressed as number (percentage) unless indicated

^†^Chi square test of homogeneity unless indicated

^^^Mann–Whitney U test

^§^ANOVA F test

Eleven infants were initially put onto the same Extensively Hydrolysed Formula (EHF, Nutramigen, Mead Johnson), two of these then progressed onto an Amino Acid Formula (AAF, Neocate, Nutricia) as their symptoms did not improve on the EHF. Two infants had already been commenced onto a soya infant formula (Wysoy, Nutricia) by their General Practitioner. From 26 weeks, all infants consuming EHF were changed to an extensively hydrolysed follow-on formula. In the control group, 16 infants consumed a follow on formula from 26 weeks onwards, whilst 10 remained on their standard formula.

**Fig. 1:**
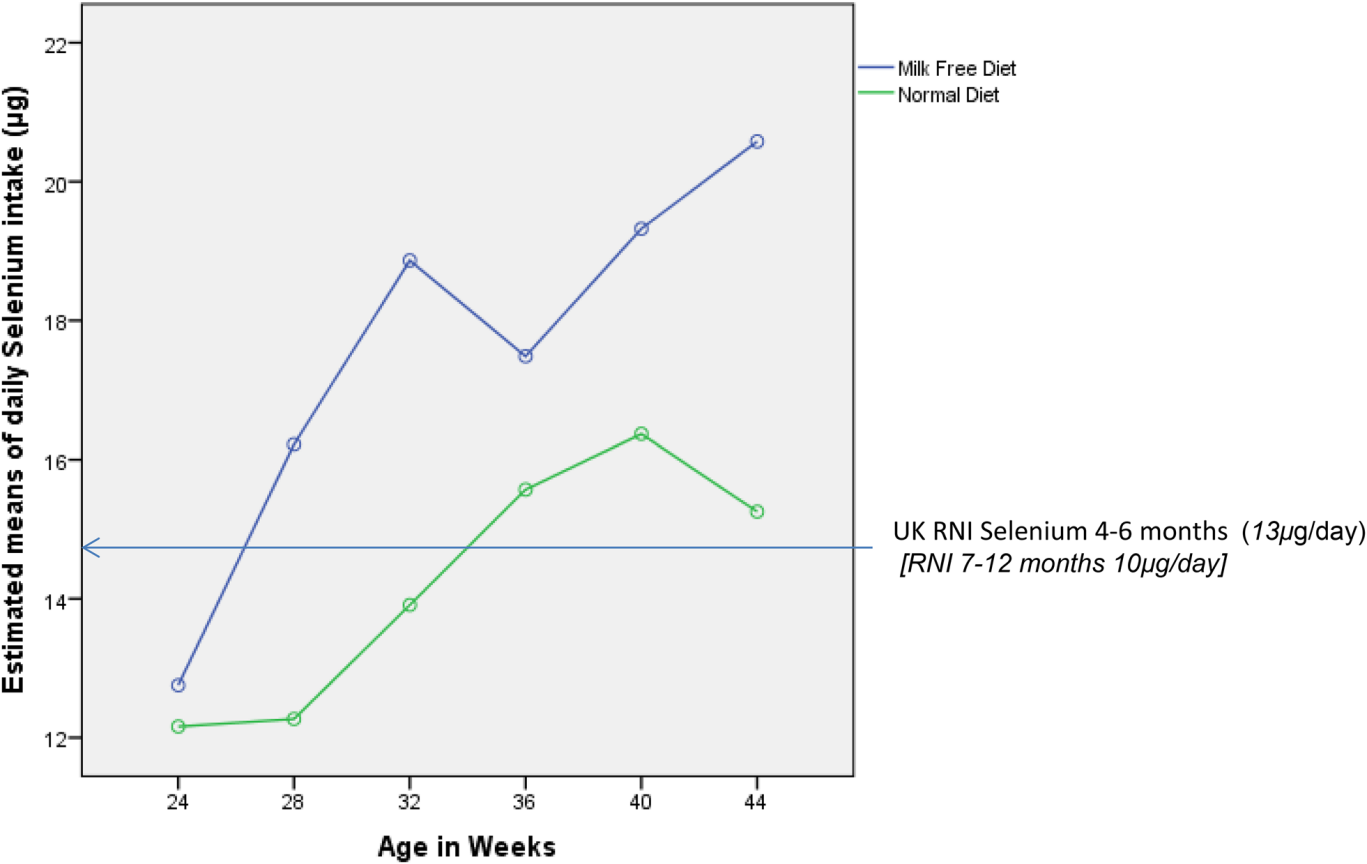
Estimated means for daily selenium intake (μg)

**Fig. 2:**
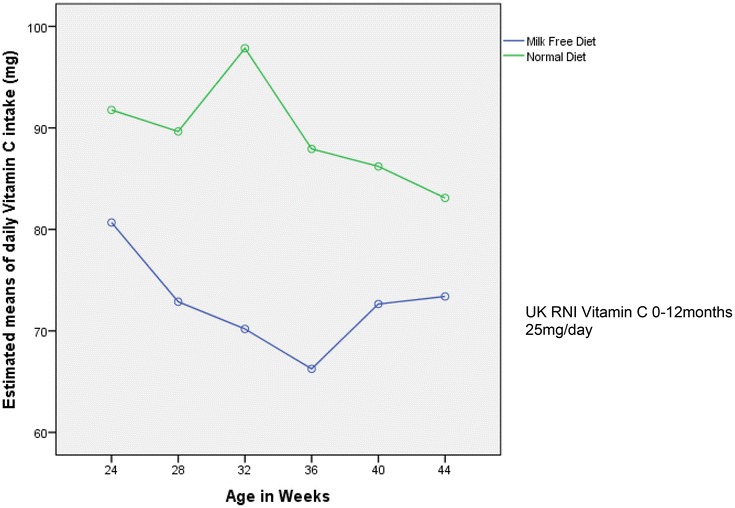
Estimated means for daily Vitamin C intake (mg)

All infants had mean intakes in excess of the requirements for energy and the recommended intakes for protein, calcium, iron, selenium, zinc, vitamins A, C, D and E. RM-ANOVA ‘between subject’ analysis indicated that the mean daily intake differed significantly between the groups across the whole time period for selenium (p = 0.003) and vitamin C (p = 0.01) (shown in Figs. 1, 2). At all time-points, selenium intake was higher for infants following a milk free diet than for infants following an unrestricted diet (p = 0.003).

Observed vitamin C intake decreased for both groups from the start of the 20 week period (24 weeks of age) compared to the end (44 weeks of age) and was higher for infants following an unrestricted diet than for infants following a milk free diet at all-time points (p = 0.001).

Differences were also found between the two study groups at differing time periods for protein, calcium, iron and vitamin E. A summary of significant differences between groups is shown in Table 2.

**Table 2:** Time points between which there was a significant difference in nutrient intake between food allergic milk-free and matched control infants and nature of the difference observed

Nutrient	Age between specific time points (weeks)	p value	Nature of difference in intake
Protein	28–32	p = 0.039	Intake higher in milk-free infants compared to control infants between these weeks
Fat	32–36	p = 0.023	Intake increases in milk-free infants at a greater rate than intake in control infants between these weeks
Calcium	36–40	p = 0.025	Intake decreases in milk-free infants but increases in control infants between these weeks
Iron	24–28	p = 0.028	Intake increases slightly in milk-free infants but increases sharply in control infants between these weeks
Selenium	24–28	p = 0.049	Intake increases dramatically in milk-free infants but only slightly in control infants between these weeks
Vitamin E	32–36	P = 0.044	Intake increases dramatically in milk-free infants but decreases slightly in control infants between these weeks

## Discussion

This study aimed to compare the nutritional intake of a group of infants consuming a cows’ milk free diet to a matched control group of infants consuming an unrestricted diet over a period of 5 months. All participants had mean dietary intakes in excess of the recommended levels (with the exception of vitamin D at age 44 weeks) and this is in agreement with data from the UK Diet and Nutrition Survey of Infants and Young Children (DNSIYC) [39]. Whilst it is reassuring that both groups of infants met their requirements for most nutrients at all time points, it must be highlighted that the majority of infants in this study were born to well-educated mothers, who may be more likely to follow recommended feeding advice than less well-educated mothers [40].

It is well known that some parents may implement restricted diets without medical supervision [10] and previous research suggests that that infants consuming exclusion diets who had not received nutritional advice were likely to have diets deficient in vitamin D and calcium compared to those who had received nutritional advice [24]. A recent study from Italy [30] confirmed that dietetic input has a positive significant effect on anthropometric and laboratory biomarkers of nutritional status in young children with CMA. In this study cows’ milk avoidance advice was provided by a specialist allergy dietitian, including timely advice to encourage a varied diet, which may have helped prevent fussy eating and feeding problems. Therefore our findings cannot be extrapolated to infants not receiving individualised dietetic advice.

Since this study collected nutritional intake data from diet diaries completed prospectively, the diaries were re-examined post hoc to collect information on the actual foods eaten to further explain the observed results. The higher selenium and vitamin C intake for infants consuming a milk exclusion diet can be explained by the use of soya products as a dairy alternative. Compared to dairy based fruit yogurts, which contributed over 50 % of the daily vitamin C intake in the control group, the soya desserts eaten by the milk free group did not generally contain fruit and therefore little if any, vitamin C. The intake of fruit as a finger food increased in the milk free group from 36 weeks of age and this explains the increase in vitamin C in the diets of these children from this time-point (Fig. 2). This increase was not seen in the control group, as their finger foods mainly consisted of milk containing foods (e.g. biscuits). The inclusion of biscuits as a regular weaning food may have implications for future preferences for sugary snack foods. It has been shown that those who consumed milk exclusion diets in infancy have lower preference for dairy foods such as chocolate and ice cream in later childhood [41].

Infants in the control group had a higher fat intake than the milk-free group at all time points, although this difference did not reach statistical significance. This is likely to be due to the inclusion of full fat dairy products in the diets of the control group. However, mean daily vitamin E intake (a fat-soluble vitamin) was broadly similar between the two groups until week 32. After this time-point, intake increased noticeably in the milk-free group, possibly due to the relatively rich vitamin E content of soya products. Of note, two previous studies [20, 24] have also reported that children with food allergies consume more vitamin E than controls. This may be due to a recommendation to include vegetable oil as a non-dairy source of fat and calories in children with multiple food allergies [42].

Statistical analysis showed mean daily iron intake to be significantly higher in the milk-free group compared to the control group between weeks 24 and 28 (p = 0.028), which can be attributed to the higher iron content of specialised formula used for CMA, compared to standard infant formula. Infants in the milk-free group transitioned to the “follow on” version of the specialised formula at age 26 weeks, under the guidance of the dietitian. The transition to follow on formula in the control group tended to occur at >26 weeks, as they were not prompted to change by a dietitian. Similar to our results, Meyer et al. [29] reported that intake of hypoallergenic formula was correlated to micronutrient intake in a group of children with food protein induced gastrointestinal allergy.

The significant difference in mean daily calcium intake between the two groups between 36 and 40 weeks can be attributed to a decline in formula intake. A decline in formula intake was seen in both groups, but infants aged between 4 and 11 months in the UK on an unrestricted diet consume between 53 and 147 g per day of milk or milk products [39], which will compensate for the reduction in calcium intake from formula. In contrast, even though infants consuming a milk free diet may be consuming some calcium containing replacement foods, these may not be eaten in large enough quantities to compensate for the decrease in formula intake. However, it must be emphasised that all infants in the milk-free group met the RNI for calcium, with none requiring a calcium supplement. Meyer et al. [29] noted that both deficiency and over supplementation of calcium is present in children consuming exclusion diets, implying that individualised dietetic advice rather than blanket recommendation of supplementation is warranted.

Higher protein intakes were found in the milk-free group, which is likely to be due to the higher protein content of specialised infant formula used in CMA. Although the difference is not large per 100 ml (0.5 g), in younger infants when total intake can be approximately 1000 ml/day, this difference could equate to as much as 5 g protein per day.

Although there was no significant difference in vitamin D intake between the two groups at any time point, intake did fall marginally below the RNI for both groups at the age of 44 weeks. This could be explained by a decline in the volume of infant formula consumed by both groups. Only one breastfeeding mother took a vitamin D supplement and no infant took a vitamin D supplement, despite Department of Health recommendations. Interestingly, the recent Diet and Nutrition Survey of infants and Young Children (DNSIYC) (2011) [39] reported that although only 7 % of those aged 7–9 months and 8 % of those aged 10–11 months took a multivitamin supplement, 94 % of those aged 5–11 months had 25-hydroxyvitamin D (25-OHD) above the lower threshold for vitamin D adequacy.

A major and unique strength of the study is that the dietary information was collected prospectively, which eliminates any recall bias, an inherent error in other dietary assessment methods. A further strength of the study is that food diaries were collected for each infant on a monthly basis. The main limitation of the study is whether the finding that a milk free diet can meet nutritional requirements can be applied to infants who have not seen a dietitian for exclusion advice. Additionally, since the data set is relatively small, there is potential for sampling error and response bias, but as the data is prospective and longitudinal, this potential is reduced. Overall, the sample size of 39 is comparable to other published studies of dietary intake in CMA [21, 22, 28]. However, it was not a randomised study and so results cannot be considered causal, but matching of the milk-free infants with controls, means the observed differences between the groups is likely to be due to the different diets rather than confounding variables.

## Conclusion

This study demonstrates that infants consuming a milk-free diet have a nutritional intake that is significantly different to matched controls consuming an unrestricted diet, but the difference is not constant and it is not seen for all nutrients. Most of the differences are a consequence of the dairy alternatives included in the milk free diet at the recommendation of the specialist allergy dietitian. However, since the main carers of all the infants following a milk-free diet received advice from a specialist allergy dietitian, these observations cannot necessarily be applied to the general population since this level of support is not always widely available. Further research is needed to explore the nutritional implications of unsupervised cows’ milk exclusion diets. However in the interim, it is important to continue to emphasise to parents and carers of infants not to restrict a child’s diet without adequate medical or dietetic intervention.

## Abbreviations

CMA: cows’ milk allergy
DNSIYC: Diet and Nutrition Survey of Infants and Young Children
EAR: estimated average requirement
EHF: extensively hydrolysed formula
PIFA study: Prevalence of Infant Food Allergy study
RNI: reference nutrient intake

## References

[1] Venter C, Pereira B, Grundy J, Clayton CB, Roberts G, Higgins B (2006). Incidence of parentally reported and clinically diagnosed food hypersensitivity in the first year of life. J Allergy Clin Immunol.

[2] Schoemaker AA, Sprikkelman AB, Grimshaw KE, Roberts G, Grabenhenrich L, Rosenfeld L (2015). Incidence and natural history of challenge-proven cow’s milk allergy in European children–EuroPrevall birth cohort. Allergy.

[3] Grimshaw KEC, Bryant T, Oliver EM, Martin J, Maskell J, Kemp T (2016). Incidence and risk factors for food hypersensitivity in UK infants: results from a birth cohort study. Clin Transl Allergy.

[4] Luyt D, Ball H, Makwana N, Green MR, Bravin K, Nasser SM (2014). BSACI guideline for the diagnosis and management of cow’s milk allergy. Clin Exp Allergy.

[5] Venter C, Laitinen K, Vlieg-Boerstra B (2012). Nutritional Aspects in diagnosis and management of food hypersensitivity—the dietitians role. J Allergy.

[6] Rona RJ, Keil T, Summers C, Gislason D, Zuidmeer L, Sodergren E (2005). The prevalence of food allergy: a meta-analysis. J Allergy Clin Immunol..

[7] Elizur A, Cohen M, Goldberg MR, Rajuan N, Katz Y (2013). Mislabelled cow’s milk allergy in infants: a prospective cohort study. Arch Dis Child.

[8] Pawankar R, Canonica G, Holgate S, Lockey P (2011). White book on allergy.

[9] Stein K (2014). Severely restricted diets in the absence of medical necessity: the unintended consequences. J Acad Nutr Diet.

[10] Eggesbø M, Botten G, Stigum H (2001). Restricted diets in children with reactions to milk and egg perceived by their parents. J Pediatr.

[11] Sinagra JL, Bordignon V, Ferraro C, Cristaudo A, Di Rocco M, Amorosi B (2007). Unnecessary milk elimination diets in children with atopic dermatitis. Pediatr Dermatol.

[12] Bergmann MM, Caubet J-C, McLin V, Belli DC, Schäppi MG, Eigenmann PA (2014). Common colic, gastroesophageal reflux and constipation in infants under 6 months of age do not necessitate an allergy work-up. Pediatr Allergy Immunol.

[13] World Health Organization. Implementation plan on maternal, infant and child nutrition. 2014. http://www.who.int/nutrition/publications/CIP_document/en/. Accessed 4 May 2016.

[14] Le Louer B, Lemale J, Garcette K, Orzechowski C, Chalvon A, Girardet J-P (2014). Severe nutritional deficiencies in young infants with inappropriate plant milk consumption. Arch Pediatr.

[15] Nwaru BI, Takkinen HM, Kaila M, Erkkola M, Ahonen S, Pekkanen J (2014). Food diversity in infancy and the risk of childhood asthma and allergies. J Allergy Clin Immunol.

[16] Roduit C, Frei R, Depner M, Schaub B, Loss G, Genuneit J (2014). Increased food diversity in the first year of life is inversely associated with allergic diseases. J Allergy Clin Immunol.

[17] Nurmatov U, Devereux G, Sheikh A (2011). Nutrients and foods for the primary prevention of asthma and allergy: systematic review and meta-analysis. J Allergy Clin Immunol.

[18] Vieira MC, Morais MB, Spolidoro JVN, Toporovski MS, Cardoso AL, Araujo GTB (2010). A survey on clinical presentation and nutritional status of infants with suspected cow’ milk allergy. BMC Pediatr.

[19] Agostoni C, Fiocchi A, Riva E, Terracciano L, Sarratud T, Martelli A (2007). Growth of infants with IgE-mediated cow’s milk allergy fed different formulas in the complementary feeding period. Pediatr Allergy Immunol.

[20] Flammarion S, Santos C, Guimber D, Jouannic L, Thumerelle C, Gottrand F (2011). Diet and nutritional status of children with food allergies. Pediatr Allergy Immunol.

[21] Tiainen J (1995). Diet and nutritional status in children with cow’s milk allergy. J Clin Nutr.

[22] Henriksen C, Eggesbø M, Halvorsen R, Botten G (2000). Nutrient intake among two-year-old children on cows’ milk-restricted diets. Acta Paediatr.

[23] Jensen VB, Jorgensen IM, Rasmussen KB, Molgaard C, Prahl P (2004). Bone mineral status in children with cow milk allergy. Pediatr Allergy Immunol.

[24] Christie L, Hine RJ, Parker JG, Burks W (2002). Food allergies in children affect nutrient intake and growth. J Am Diet Assoc.

[25] Devlin J, Stanton RH, David TJ (1989). Calcium intake and cows’ milk free diets. Arch Dis Child.

[26] Mabin DC, Sykes AE, David TJ (1995). Nutritional content of few foods diet in atopic dermatitis. Arch Dis Child.

[27] Noimark L, Cox HE (2008). Nutritional problems related to food allergy in childhood. Pediatr Allergy Immunol.

[28] Berry MJ, Adams J, Voutilainen H, Feustel PJ, Celestin J, Järvinen KM (2015). Impact of elimination diets on growth and nutritional status in children with multiple food allergies. Pediatr Allergy Immunol.

[29] Meyer R, De Koker C, Dziubak R, Godwin H, Dominguez-ortega G, Shah N (2014). Dietary elimination of children with food protein induced gastrointestinal allergy—micronutrient adequacy with and without a hypoallergenic formula?. Clin Transl Allergy.

[30] Berni Canani R, Leone L, D’Auria E, Riva E, Nocerino R, Ruotolo S (2014). The effects of dietary counseling on children with food allergy: a prospective, multicenter intervention study. J Acad Nutr Diet.

[31] Andersen LF, Lande B, Trygg K, Hay G (2004). Validation of a semi-quantitative food-frequency questionnaire used among 2-year-old Norwegian children. Public Health Nutr..

[32] Keil T, McBride D, Grimshaw K, Niggemann B, Xepapadaki P, Zannikos K (2010). The multinational birth cohort of EuroPrevall: background, aims and methods. Allergy Eur J Allergy Clin Immunol.

[33] Grimshaw KEC, Maskell J, Oliver EM, Morris RCG, Foote KD, Mills ENC (2013). Introduction of complementary foods and the relationship to food allergy. Pediatrics.

[34] Grimshaw KEC, Maskell J, Oliver EM, Morris RCG, Foote KD, Mills ENC (2014). Diet and food allergy development during infancy: birth cohort study findings using prospective food diary data. J Allergy Clin Immunol.

[35] Sicherer SH (2001). Clinical implications of cross-reactive food allergens. J Allergy Clin Immunol.

[36] Paul AA, Black AE, Evans J, Cole TJ, Whitehead RG (1988). Breastmilk intake and growth in infants from two to ten months. J Hum Nutr Diet.

[37] Haisma H, Coward WA, Albernaz E, Visser GH, Wells JCK, Wright A (2003). Breast milk and energy intake in exclusively, predominantly, and partially breast-fed infants. Eur J Clin Nutr.

[38] Department of Health. Dietary reference values for food energy and nutrients for the United Kingdom: report of the panel on dietary reference values of the committee on mediacla aspects of food policy. Her Majesty's Stationery Office; 1991. 1961974

[39] Lennox A, Sommerville J, Ong K, Henderson H, Allen R. Diet and nutrition survey of infants and young children 2011. A survey carried out on behalf of the Department of Health and Food Standards Agency. 2013.

[40] Robinson S, Marriott L, Poole J, Crozier S, Borland S, Lawrence W (2007). Dietary patterns in infancy: the importance of maternal and family influences on feeding practice. Br J Nutr.

[41] Maslin K, Jane G, Glasbey G, Dean T, Arshad SH, Grimshaw KEC (2016). Cows’ milk exclusion diet during infancy: Is there a long term effect on children’s eating behaviour and food preferences?. Pediatr Allergy Immunol.

[42] Sova C, Feuling MB, Baumler M, Gleason L, Tam JS, Zafra H (2013). Systematic review of nutrient intake and growth in children with multiple IgE-mediated food allergies. Nutr Clin Pract..

